# The ten amino acids of the oxygen-evolving enhancer of tobacco is sufficient as the peptide residues for protein transport to the chloroplast thylakoid

**DOI:** 10.1007/s11103-020-01106-8

**Published:** 2021-01-03

**Authors:** Sang Hoon Ma, Hyun Min Kim, Se Hee Park, Seo Young Park, Thanh Dat Mai, Ju Hui Do, Yeonjong Koo, Young Hee Joung

**Affiliations:** 1 School of Biological Science and Technology, Chonnam National University, Gwangju, 61186 South Korea; 2 Department of Agricultural Chemistry, Chonnam National University, Gwangju, 61186 South Korea

**Keywords:** Chloroplast, Tat pathway, Thylakoid lumen, Transit peptides

## Abstract

**Key message:**

The thylakoid transit peptide of tobacco oxygen-evolving enhancer protein contains a minimal ten amino acid sequences for thylakoid lumen transports. This ten amino acids do not contain twin-arginine, which is required for typical chloroplast lumen translocation.

**Abstract:**

Chloroplasts are intracellular organelles responsible for photosynthesis to produce organic carbon for all organisms. Numerous proteins must be transported from the cytosol to chloroplasts to support photosynthesis. This transport is facilitated by chloroplast transit peptides (TPs). Four chloroplast thylakoid lumen TPs were isolated from *Nicotiana tabacum* and were functionally analyzed as thylakoid lumen TPs. Typical chloroplast stroma-transit peptides and thylakoid lumen transit peptides (tTPs) are found in *N. tabacum* transit peptides (NtTPs) and the functions of these peptides are confirmed with TP–GFP fusion proteins under fluorescence microscopy and chloroplast fractionation, followed by Western blot analysis. During the functional analysis of tTPs, we uncovered the minimum 10 amino acid sequence is sufficient for thylakoid lumen transport. These ten amino acids can efficiently translocate GFP protein, even if they do not contain the twin-arginine residues required for the twin-arginine translocation (Tat) pathway, which is a typical thylakoid lumen transport. Further, thylakoid lumen transporting processes through the Tat pathway was examined by analyzing tTP sequence functions and we demonstrate that the importance of hydrophobic core for the tTP cleavage and target protein translocation.

**Supplementary Information:**

The online version of this article (10.1007/s11103-020-01106-8) contains supplementary material, which is available to authorized users.

## Introduction

Chloroplasts are responsible for photosynthesis and the production of important biomolecules, such as lipids, amino acids, and hormones, which are essential for plant development and physiology (Jarvis and Robinson 2004; Pfannschmidt and Yang 2012). Chloroplasts are structurally complex organelles, enclosed by a double membrane referred to as outer and inner envelopes. Chloroplasts also display a third internal membrane called thylakoid membrane. The membrane system produces three compartments: the intermembrane space, stroma, and thylakoid lumen (Keegstra and Cline 1999; Jarvis and Robinson 2004; Soll and Schleiff 2004). Transcription and translation processes and metabolic reactions, such as the Calvin cycle, occur in the stroma. Photosynthesis occurs in the thylakoid lumen, with the production of NADPH, ATP, and oxygen (Pfannschmidt and Yang 2012).

Chloroplasts contain their own genome, which are about 120–170 kb in size, coding for 100–120 genes that encode proteins required for transcription and translation in the stroma and photosynthesis in the thylakoid lumen (Downie and Palmer 1992; Jarvis and Robinson 2004; Soll and Schleiff 2004; Shaw et al. 2007; Inaba and Schnell 2008). Despite possessing their own genome, the vast majority of proteins in chloroplasts are nuclear-encoded, synthesized as precursor proteins in the cytoplasm and transported to the chloroplast. For example, in *Arabidopsis*, approximately 2100–3600 proteins are synthesized in the cytosol and imported into plastids (Abdallah et al. 2000; Leister 2003; Jarvis 2004; Inaba and Schnell 2008). The transport of nuclear-encoded proteins requires specialized transit peptides (TPs) that typically exist in N-terminal regions of imported proteins (Soll and Schleiff 2004; Jarvis 2008). TPs guide chloroplast proteins to translocate into the stroma and are removed after translocation (Keegstra et al. 1989; Soll and Schleiff 2004; Jarvis 2008).

TPs are necessary for the import of nuclear-encoded proteins into chloroplasts. TP sequences vary greatly, depending on the protein and plant species, but several characteristic features of TPs are reported. They vary in residue lengths and tend to form helices under hydrophobic conditions. TPs can be divided into three domains, the N-terminal, central, and C-terminal regions, each with its own characteristics. The N-terminal region is uncharged, whereas the C-terminal region is enriched with arginine residues. The central region displays hydroxylated and charged amino acids (Keegstra et al. 1989; Claros et al. 1997; Bruce 2000, 2001).

Nuclear-encoded proteins with TPs are imported into chloroplasts by translocons on the outer chloroplast membrane and other translocons at the inner chloroplast membrane (Kouranov et al. 1998; Inaba et al. 2005; Chen and Li 2007; Inaba and Schnell 2008; Jarvis 2008; Lung and Chuong 2012). TPs on proteins that reach the stroma are removed by stromal processing peptidase (SPP). Unlike TPs of stromal proteins, TPs of nuclear-encoded thylakoid proteins are removed from chloroplasts in a different manner. They are first removed from the stroma by SPP, and the remaining protein is processed after passing through the thylakoid membrane by thylakoid processing peptidase (TPP). Thus, the transport of thylakoid proteins requires a two-step import system with two separate TPs. Chloroplast transit peptide (cTP) is required in the transport of thylakoid proteins from cytosol to stroma, and thylakoid transit peptide (tTP) is required for import to thylakoid lumen. After localization, cTP and tTP are removed from nuclear-encoded proteins (Smeekens et al. 1986; Smeekens and Weisbeek 1988; Robinson and Klösgen 1994; Soll and Schleiff 2004; Jarvis 2008; Teixeira and Glaser 2013).

Most proteins involved in photosynthesis are thylakoid proteins that use a two-step import process. Import across the thylakoid membrane may involve four distinct transport pathways: twin-arginine translocation (Tat), secretion (Sec), signal recognition particle (SRP), and spontaneous. The pathways involved in the transport of the thylakoid lumen protein are Sec and Tat, whereas the pathways for the insertion of thylakoid membrane proteins are SRP and spontaneous (Jarvis and Robinson 2004; Gutensohn et al. 2006; Cline and Theg 2007; Jarvis 2008; Aldridge et al. 2009). Sec and SRP pathways translocate proteins in an unfolded state into the thylakoid lumen and require the activity of chaperone proteins to prevent protein folding. In addition, the Sec and SRP pathways obtain energy for translocation through hydrolysis of nucleoside triphosphates (NTPs), ATP in the case of Sec, and GTP in the case of SRP. The Tat pathway translocates protein of a folded state by recognizing the twin-arginine motif upstream of hydrophobic residues in the TP. Also, the Tat pathway only requires the transthylakoid proton gradient (ΔpH), not the energy as ATP or GTP (Cline et al. 1992; Robinson et al. 2001).

The Tat pathway includes integrating membrane proteins Hcf106, cpTatC, and Tha4 (Settles et al. 1997; Mori et al. 1999; Walker et al. 1999; New et al. 2018). These proteins are present in the thylakoid membrane as two subcomplexes: Hcf106 and cpTatC form the receptor complex, and Tha4 forms an oligomeric complex (Cline and Mori 2001; Mori and Cline 2002; Dabney-Smith et al. 2006). In the receptor complex, cpTatC and Hcf106 interact with different regions of the TP. The cpTatC is strongly bound to residues immediately adjacent to the twin-arginine motif, and Hcf106 is less strongly bound to residues of the hydrophobic core and early mature protein (Cline and Mori 2001; Gohlke et al. 2005).

The Tat pathway is roughly divided into several steps. First, precursor protein binds via TP to the cpTatC–Hcf106 receptor complex in the thylakoid membrane. Sequentially, the receptor complex bound to precursor protein is assembled with Tha4 oligomers, and a putative translocase is formed. The precursor protein is transported across the thylakoid membrane using the ΔpH gradient, and mature protein with TP removed by TPP is released into the thylakoid lumen (Celedon and Cline 2013).

Thylakoid proteins, such as oxygen-evolving enhancer protein complex (OE), plastocyanin, and the light-harvesting chlorophyll a/b proteins, are almost always encoded in the nucleus (Jarvis and Robinson 2004; Jarvi et al. 2013). A representative thylakoid protein is OE, consisting of three proteins (PsbO, PsbP, and PsbQ), which helps stabilize and optimize the water-splitting reactions in photosystem II (Seidler 1996; Hankamer et al. 1997; De Las et al. 2007; Jarvi et al. 2013). OE is a typical translocated thylakoid protein and has been used extensively for the study of protein translocation mechanisms. In this study, we identified stromal and thylakoid transit peptides from tobacco OE23. We investigated the peptide functions by monitoring the GFP protein transport within the chloroplast. We used serial deletion of thylakoid TPs to help dissect Tat pathway-mediated thylakoid translocation. The results increase our understanding of protein transport within chloroplasts.

## Results

### OE23 TPs have typical lumen targeting peptide sequences

**Fig. 1:**
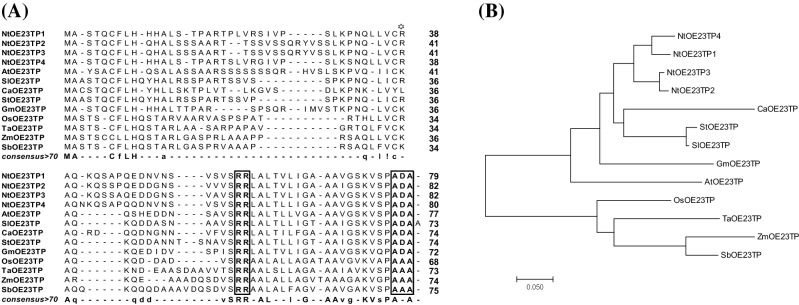
Comparison of plant *OE23* chloroplast targeting sequences. **a** Sequence comparison using Clustal Omega. Open star indicates the residue expected to cleave during chloroplast outer membrane translocation, and square boxes indicate conserved residues of TP. **b** The phylogenic tree of plant *OE23* chloroplast targeting sequences. Tree drawing was prepared using the neighbor-joining method based on the Poisson correction model in the MEGA7 program. Nt, *Nicotiana tabacum*; Ca, *Capsicum annuum*; St, *Solanum tuberosum*; Sl, *Solanum lycopersicum*; Gm, *Glycine max*; At, *Arabidopsis thaliana*; Os, *Oryza sativa*; Ta, *Triticum aestivum*; Zm, *Zea mays*; Sb, *Sorghum bicolor*; TP, transit peptide

The four TPs coded by the *NtOE23* gene were isolated from the genomic DNA of *N. tabacum* cv. Xanthi-nc and termed TP1, TP2, TP3, and TP4. Bioinformatic prediction using the ChloroP 1.1 program (http://www.cbs.dtu.dk/services/ChloroP/) identified 39 amino acids (TP1 and TP4) or 42 amino acids (TP2 and TP3) from N-terminals as probable chloroplast transit peptides, cTP, and the subsequent peptides were expected to be thylakoid membrane transit peptides, tTP (Fig. 1a). Multiple sequence alignment of putative cTP and tTP of NtOE23s using Clustal Omega revealed cleavage sites: an L-V-C-R sequence of cTP region and a twin-arginine (RR)-AxA sequences of tTP region were highly conserved in all NtOE23s (Fig. 1a). Amino acid sequence identity and similarity among the four NtOE23 TPs (TP1, 2, 3, and 4) were quantified using the EMBOSS Needle program (https://www.ebi.ac.uk/Tools/psa/emboss_needle/). TP1 exhibits the highest similarity and identity with TP4, whereas TP2 exhibits the highest similarity and identity with TP3 (Supplementary Table S1).

The TPs of *OE23* in diverse plants, such as *Capsicum annuum*, *Solanum tuberosum*, *Solanum lycopersicum*, *Glycine max*, *Arabidopsis thaliana*, *Oryza sativa*, *Triticum aestivum*, *Zea mays*, and *Sorghum bicolor*, were also highly conserved and reflect functional identity as thylakoid lumen TPs (Fig. 1). Bioinformatics revealed that the NtOE23 TPs were highly conserved in the OE23 TPs of Solanaceae family plants (identity: 57.3–67.4%, similarity: 67.5–74.7%) compared with the OE23 TPs of Poaceae family plants (identity: 43.0–50.0%, similarity: 54.9–59.3%) (Supplementary Table S2). These findings suggest that the NtOE23 TPs were more closely related to the OE23 TPs of Solanaceae family plants, rather than the OE23 TPs of Poaceae family plants. The phylogenetic tree built from the alignment supported this finding (Fig. 1b). The four TPs of NtOE23 have the conserved sequences, RR and A×A residues for thylakoid lumen translocation through the Tat pathway, as do proteins of different plant species (Supplementary Figure S1A).

### NtOE23 TPs are sufficient for chloroplast localization of the target gene

**Fig. 2:**
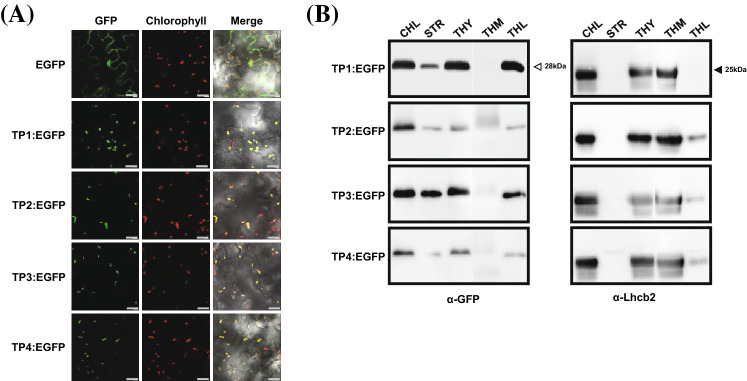
The chloroplast lumen localization of NtOE23TPs. **a** The fusion constructs were agro-infiltrated into *N. benthamiana* leaves. Red indicates chlorophyll autofluorescence. The green fluorescent signals were co-localized with the red fluorescent signals of chlorophyll (merge). Scale bars, 20 μm. **b** Localization of TP fused with GFP proteins. Chloroplast proteins were separately collected from the chloroplast compartments, and Western blotting analysis was conducted using anti-GFP antibody. Lhcb2 signal is used for confirming the separation of thylakoid membrane (THM) with stroma (STR) and thylakoid lumen (THL) in the chloroplast fractionations. TSP, CHL, and THY represent total soluble protein, chloroplast fraction, and thylakoid fraction, respectively

Subcellular localization of the TP–GFP proteins was observed by transient expression in tobacco leaves (Fig. 2a). The four TP-directed GFP signals clearly overlap with chlorophyll fluorescence. Enrichment of TP–GFP proteins was analyzed by separating stromal/thylakoid and thylakoid membrane/lumen fractions through serial centrifugation (Fig. 2b). The distribution of the LHCII type II chlorophyll a/b-binding protein (Lhcb2) was analyzed as an internal marker of fractionation. Lhcb2 localized in the thylakoid membrane (THM) and the four TP peptides translocated GFP protein primarily to the thylakoid lumen (THL). Comparison of the GFP band intensities between the stroma and thylakoid (STR and THR) revealed that TP1 and TP4 peptides transported GFP into the THL in higher proportion, but TP2 and TP3 peptides transported GFP protein to stroma and thylakoid in uniform proportion. This result indicates that TP1 and TP4 are more efficient than TP2 and TP3 as thylakoid transit peptides, which is consistent with the sequence analysis using EMBOSS Needle program in Supplementary Table S1. From the GFP image and Western blotting results, we conclude that *OE23* TPs are sufficient for the transport of nuclear-coded protein and that all four TPs have similar functional activity for transport proteins into the THL.

**Fig. 3:**
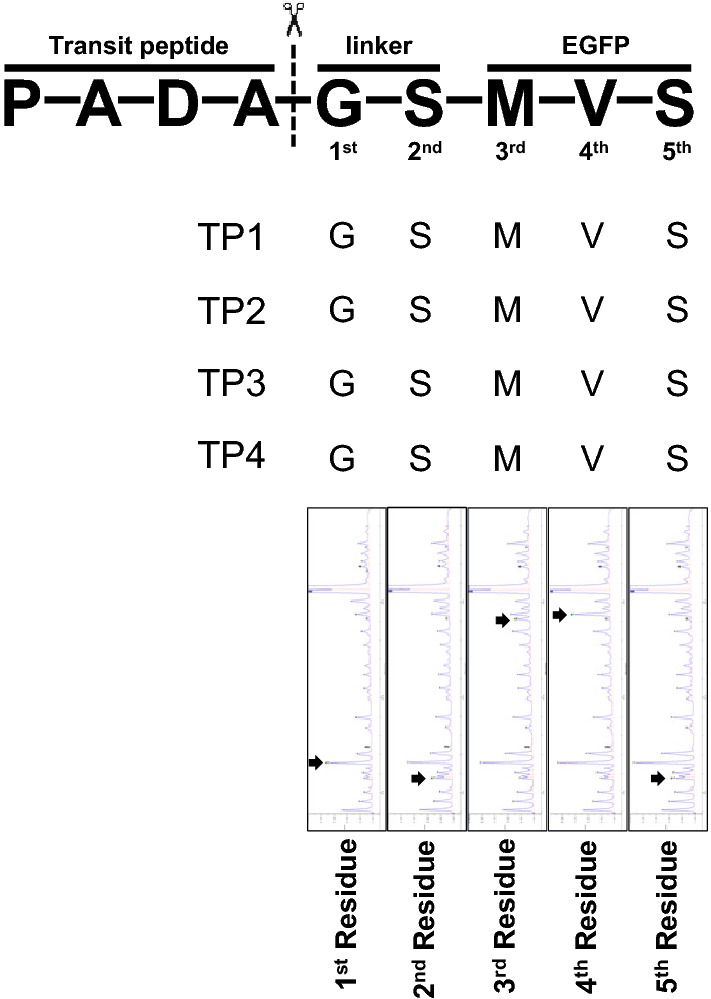
Transit peptide cleaves of NtOE23TPs. The five N-terminal sequences were detected via Edman sequencing. Arrows indicate the probable peak of the identified amino acid residue

According to Jarvis (2008), TP was cleaved during translocation in the THM. To confirm the cleavage of NtOE23 TPs, Edman sequencing was performed using the total soluble protein (TSP) fractions isolated from the transgenic plant leaves to identify the cleavage site of TP–GFP protein. A G–S linker sequence was followed by a M–V–S GFP N-terminal sequence in all four TPs, indicating that tTP was cleaved exactly after A–D–A sequences (Fig. 3). This result indicates that TPs are cleaved during chloroplast translocation.

### cTP1 is essential for chloroplast localization

**Fig. 4:**
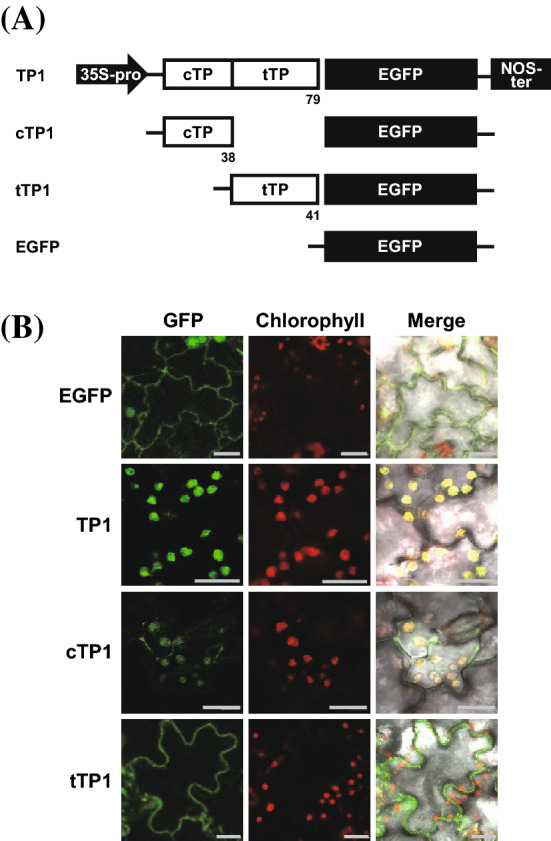
The functional comparison between cTP1 and tTP1 peptides for GFP chloroplast targeting. **a** Schematic representation of the EGFP fusion constructs with the truncated domain of TP1 for transformation. **b** Fluorescence images of the subcellular localization of green EGFP signals directed by cTP1 and tTP1 (GFP lane), the red chlorophyll autofluorescence (chlorophyll lane), and co-localization of green and red signals (merge) are presented. Scale bars, 20 μm

Intact TPs transported GFP to the THL (Fig. 2a). Further, TP1 peptides were divided into cTP1 and tTP1 domains, and cTP1–GFP or tTP1–GFP constructs were transiently expressed in tobacco leaves (Fig. 4a). Only cTP1–GFP succeeds in transporting GFP to chloroplasts (third panel in Fig. 4b) like a whole TP containing construct (second panel in Fig. 4b). Since tTP1–GFP failed to translocate GFP into the chloroplast, we conclude that cTP1 is essential as a chloroplast TPs. The GFP transport efficiency between cTP1–GFP and whole TP1–GFP was not different in the GFP image analysis or intensity in the chloroplast. Therefore, we concluded the cTP1 is sufficient for transporting proteins to chloroplasts.

### RR and A×A sequences of tTP1 are necessary for efficient tTP1 peptide cleavage

**Fig. 5:**
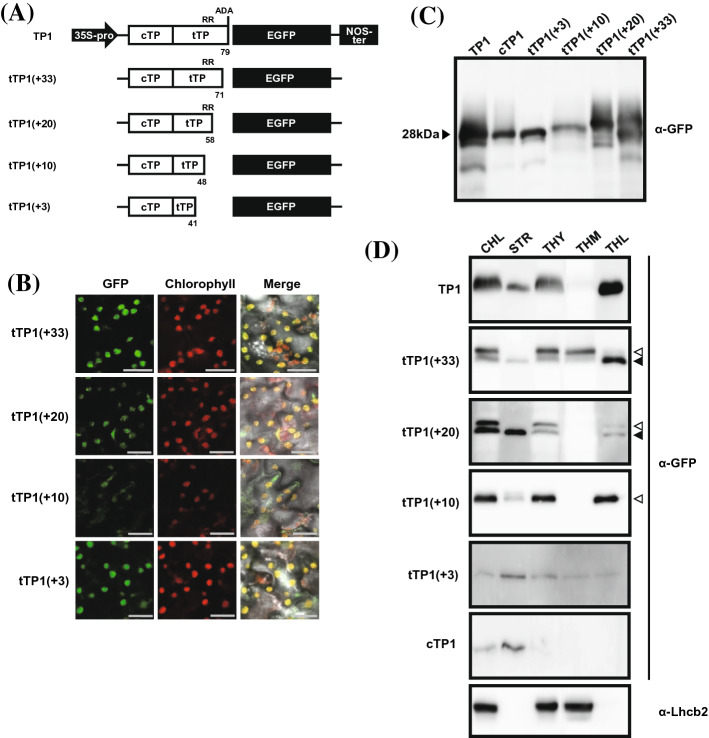
The functional study of tTP1 peptide by serial deletion. **a** Construction of recombinant plasmids carrying EGFPs fused with truncated tTP1. **b** Images are showing the fluorescence signals of EGFP directed by truncated tTP1s (GFP) and autofluorescence of chlorophyll to observe chlorophyll localization of TPs–EGFPs. Scale bars, 20 μm. **c** The protein size comparison of TPs–EGFPs extracted from the indicated transgenic plants. **d** Stroma and thylakoid of the chloroplast are extracted separately to determine the sub-organelle localization of TPs–EGFPs. Localization of Lhcb2 is indicated as a control of sub-organelle extractions. Lhcb2 visualization was performed for all panels repeatedly, and the same results as the representative were obtained. The black arrowheads and white arrowheads indicate the GFP proteins that cleaved and uncleaved their signal peptides, respectively. CHL, chloroplast; STR, stroma; THY, thylakoid; THM, thylakoid membrane; THL, thylakoid lumen

GFP was fused to serially deleted tTP1 on its C-terminal and fused with cTP1 on its N-terminal for chloroplast transporting (Fig. 5a) in order to investigate the function of the tTP1 region. The tTP1 region was deleted by 8, 21, 31, and 38 amino acids from the C-terminal and termed tTP1(+ 33), tTP1(+ 20), tTP1(+ 10), and tTP1(+ 3), respectively (Fig. 5a). All constructs succeeded in localizing GFP signals in the chloroplast. cTP1 peptides are expected to translocate GFP proteins into the chloroplasts (Fig. 5b).

Total cellular protein was collected, and GFP protein sizes were compared side by side in the Western blotting (Fig. 5c) in order to investigate whether TP1 cleavage depends on the tTP1 length. cTP1 revealed the same GFP protein size with TP1 on the gel, indicating that cTP1 peptides were cleaved as expected (Fig. 5c, 1st and 2nd lane, marked with arrow head, 28 kDa). However, tTP1(+ 10) exhibited a larger size of GFP than cTP1 (Fig. 5c, 4th lane). Thus, tTP1(+ 10) is not cleaved. tTP1(+ 20) and tTP1(+ 33) showed tTP1 cleavage, but the cleavage of tTP1(+ 20) was not efficient which included only the RR sequences (Fig. 5c, 5th lane and d, 3rd panel). The tTP1(+ 33) constructs included the hydrophobic core and exhibited higher efficiency for tTP1 cleavage. We conclude that the RR and A–D–A sequences are essential for tTP1 cleavage, as previously reported (Chaddock et al. 1995; Henry et al. 1997; Gérard and Cline 2006; Frielingsdorf and Klösgen 2007).

### tTP1 is essential for translocating protein to the thylakoid

Subcellular fractionation and Western blotting were conducted (Fig. 5d) to analyze sub-chloroplast GFP localization. cTP1 fused with GFP was found only in the stromal fraction. tTP1(+ 10) exhibited a significant thylakoid fraction targeting activity. Further, most GFP proteins were successfully translocated into the THL (Fig. 5d, 4th panel). tTP1(+ 10) is thus sufficient for THL translocation without Tat pathway machinery that recognizes RR residues. The tTP1(+ 10) sequence includes high hydrophilic residues from the analysis using ProtScale (Supplementary Figure S1B). Also, tTP1(+ 10) peptides were not cleaved after lumen transportation (Fig. 5c and d). tTP1(+ 20) and tTP1(+ 33) exhibited multiple GFP protein sizes (white and black arrowheads) and inefficient tTP1 peptide cleavages (as in Fig. 5c). The GFP signal intensity of the tTP1(+ 33) was much stronger in the THY fraction than in the STR fraction but the GFP signal intensity of the tTP1(+ 20) was stronger in the STR fraction than in the THY fraction. This result shows the translocation efficiency of tTP1(+ 33) to the THL was much higher than that observed for tTP1(+ 20), but much lower than that for full-length transit peptide, TP1 (Fig. 5d, 1st to 3rd panels). Interestingly, tTP1(+ 33) revealed substantial THM localization of proteins, which likely reflects hydrophobic domains between (+ 20) and (+ 33) increased interaction of target protein with THM. Therefore, we expect that RR residues are not enough to bind to Tat machinery located on the THM. Additional hydrophobic residues can enhance peptide–Tat machinery binding. Further, GFP sizes found in the stromal fraction of tTP1(+ 20) and tTP1(+ 33) were the same as those of TP1 cleaved GFP. This result represents tTP1 cleavage at the THM on the stromal side before translocation (Fig. 5d). The similar size of GFP in the stromal and thylakoid fractions were found in the other NtOE23 TPs (Fig. 2b, STR lane). These results represent stromal GFP expected from the translocation failure after TP cleavage.

**Fig. 6:**
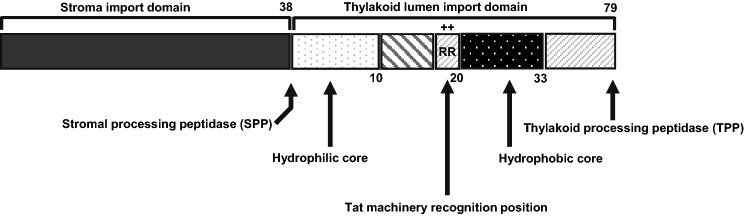
The schematic diagram of the local characterization of the NtOE23 TP1

In this study, we conclude that THL targeting TPs are functionally divided into a hydrophilic core (+ 10), a twin-arginine motif for binding with Tat machinery, a hydrophobic core for stabilizing Tat machinery binding, and AxA sequences for tTP cleavage (Fig. 6).

## Discussion

In this study, we found that the tTP1 region was specific for thylakoid localization. This result is supported by the observation that GFP proteins are localized in the cytoplasm, whereas GFP fused with NtOE23 TP1 (TP1–GFP) was localized at the THL. Western blotting of the subcellular fractions also indicated that GFP proteins with intact TP1 of NtOE23 were mainly located in the THL fraction. GFP proteins with cTP1 and truncated tTP1 were found in the stromal, THL, and even THM fractions. However, only GFP proteins with cTP1 were detected in the chloroplast and stromal fractions, not in the thylakoid.

It was previously reported that OE23 protein uses the Tat pathway to translocate into the thylakoid, and the twin-arginine motif is essential in the process (Chaddock et al. 1995; Robinson and Bolhuis 2001). The tTP regions of the four NtOE23 proteins were conserved and contained the twin-arginine motif (Fig. 1a). Therefore, NtOE23 displays typical THL transit peptides through GFP tagging and chloroplast compartment fractionation. However, our results also indicate that thylakoid localization of NtOE23 is independent of the twin-arginine motif since truncated TP1 without the twin-arginine motif (+ 10) still localized GFP protein thylakoid (Fig. 5d). The tTP1(+ 10) construct shows that this short peptide leads to target protein transport without binding to the cpTatC-Hcf106 complex that interacts with the twin-arginine motif (Settles et al. 1997; Mori et al. 1999; New et al. 2018). Further, the tTP1(+ 10) peptide is intact after lumen transportation (Fig. 5c and d). Thus, tTP1(+ 10) is expected to transport THMs through other than the Tat pathway. Two other thylakoid transport systems exist, Sec and SRP pathways. However, the Sec pathway transports only unfolded peptides, and the SRP pathway transports THM incorporating protein (Jarvis and Robinson 2004). Neither pathway explains tTP1(+ 10) transport because GFP folds very quickly (Marques et al. 2004), and SRP pathway is a thylakoid membrane targeting pathway. Therefore, we conclude that an unknown thylakoid transporting system is involved.

The results of Edman sequencing revealed that intact TP is removed after transport to the THL (Fig. 3).TPP is involved in the cleavage of TP1 of NtOE23 and is a membrane-bound serine protease, the type I signal peptidase (SPase I) family (Dalbey et al. 1997; Hsu et al. 2011; Teixeira and Glaser 2013). TPP is also a 30 kDa thylakoid processing enzyme that recognizes the Ala–X–Ala sequence of TPs (James et al. 1989; von Heijne et al. 1989; Frielingsdorf and Klösgen 2007; Teixeira and Glaser 2013). The tTP region of NtOE23 contains the twin-arginine motif and regulates thylakoid localization of target proteins (Finazzi et al. 2003; Gutensohn et al. 2006; Aldridge et al. 2009). The twin-arginine motif is confirmed to be essential for peptide cleavage, and the AxA sequence enhances cleavage However, we found that the twin-arginine motif in the tTP1 of NtOE23 is not sufficient for THL localization of GFP proteins. Further, we found that the hydrophobic region near the C-terminal of tTP is important for GFP protein localization at the THM and enhances peptide cleavage and translocation of GFP into the lumen.

In conclusion, we revealed domain-specific functions of the tTP sequence. The protein includes a hydrophilic region, a twin-arginine, and a hydrophobic region with an AxA sequence. The hydrophilic region of tTP is sufficient to transport GFP protein to the THL without peptide cleavage. Finally, the hydrophobic region of tTP locate the GFP protein in the THM and facilitates the target peptide cleavage.

## Materials and methods

### Plant growth conditions

Tobacco plants (*N. tabacum* cv. Xanthi-nc. and *Nicotiana benthamiana*) were grown in a culture room at 16 h of light and 8 h of darkness at 23 °C. From 10 to 14-week-old tobacco was used for microscopic observation and protein extraction.

### Vector constructs

The transit peptide (TP) DNA fragments of *N. tabacum* 23 kDa oxygen-evolving protein (*OE23*) were amplified with specific primer sets (Supplementary Table S3) designed according to the GenBank accession Nos. X55354, X62425, and X62427. PCR was performed at 95 °C for 10 min, followed by 35 cycles of 95 °C for 30 s, 55 °C for 30 s, and 72 °C for 30 s, with a final extension at 72 °C for 10 min using gDNA. Amplified fragments were cloned into a pBI121 vector containing an enhanced green fluorescence protein (*EGFP*) gene. Vectors with trimmed TP1 were constructed with PCR amplified fragments using specific primer sets (Supplementary Table S3) and full-length TP1 as a template. The amplified fragments replaced the TP1 fragment in the pBI121:TP1:EGFP vector. These vectors were transformed into *Agrobacterium tumefaciens*. Primer sequences are listed in Supplementary Table S3.

### Agroinfiltration of *N. benthamiana*

Agroinfiltration was performed according to a previously described method, with slight modifications (Hou et al. 2011). *Agrobacterium tumefaciens* strain GV3101 cells harboring recombinant plasmids were inoculated into YEP media containing antibiotics, 10 mM 2-(*N*-morpholino) ethanesulfonic acid (MES) (pH 5.6), and 200 uM acetosyringone. Cells were grown O/N at 28 °C with shaking and then harvested by centrifugation. Then, they were re-suspended to OD_600_ of 0.8–1.0 in 10 mM MgCl_2_ solution (pH 5.6) containing 200 uM acetosyringone. The *Agrobacterium* inoculum was left to stand at room temperature for 3 h with gentle shaking and then injected into the leaf undersides of 6-week-old *N. benthamiana* plants using a 1 mL syringe. After 3 days, infiltrated leaves were harvested, and frozen in liquid nitrogen for further analysis.

### Generation of transgenic plants

The transformation was performed with a slight modification of the *Agrobacterium*-mediated transformation using the leaf disc method described elsewhere (Horsch et al. 1985). *Agrobacterium tumefaciens* strain LBA4404 cells harboring recombinant plasmids were inoculated into YEP medium containing antibiotics and 200 μM acetosyringone and grown O/N at 28 °C with shaking. Cells were harvested by centrifugation and re-suspended in infiltration medium (2.2 g/L MS medium including vitamins, 2% sucrose, pH 5.7). Healthy tobacco leaves grown under sterile conditions were cut into 0.5 cm squares, and leaf disks were soaked in infiltration medium with cells. Leaf disks were placed on co-culture medium (4.4 g/L MS medium including vitamins, 3% sucrose, 2 mg/L 2,4-D, and 200 uM acetosyringone, pH 5.8). After 2 days of co-cultivation, disks were transferred to shoot induction medium (4.4 g/L MS medium including vitamins, 3% sucrose, 50 mg/L kanamycin, 250 mg/L cefotaxime, 0.01 mg/L NAA, 0.1 mg/L GA3, and 1 mg/L zeatin, pH 5.8) and then to fresh medium every 2 weeks. Regenerated shoots were excised from callus and transferred to root induction medium (4.4 g/L MS medium including vitamins, 1.5% sucrose, 50 mg/L kanamycin, 250 mg/L cefotaxime, and 2 mg/L NAA, pH 5.8). After the roots were regenerated from shoots, regenerated plants were transferred to soil.

### Observation of GFP fluorescence

Tobacco leaves after agro-infiltration were mounted in water under a microscope cover glass. GFP fluorescence was observed using a confocal laser scanning microscope (Leica TCS SP5/AOBS/Tandem, Germany) with excitation and emission wavelengths of 488 and 494–547 nm, respectively. Chlorophyll fluorescence was observed at emission wavelengths of 646–699 nm. The Leica Application Suite Advanced Fluorescence v4.3 software (Leica Microsystems, Germany) was used for confocal image co-localization and image analyses.

### Phylogenetic and sequence analysis

The *OE23* TP amino acid sequences of various plants were obtained from NCBI. Amino acid sequence identity and similarity were derived using the EMBOSS Needle program (https://www.ebi.ac.uk/Tools/psa/emboss_needle/); multiple sequence alignment was performed using the CLUSTAL W algorithm. Alignment results were used for phylogenetic analysis, and construction of the phylogenetic tree was performed using the MEGA 7 software (Kumar et al. 2016) via the neighbor-joining method. Hydropathy plots of TP residues were obtained from EMBOSS Pepinfo (https://www.ebi.ac.uk/services/).

### Protoplast isolation

Protoplasts were isolated from transgenic plants using a modified protocol for *A. thaliana* protoplast isolation (Somerville et al. 1981). Briefly, the leaf tissues of transgenic tobacco were cut into 2 mm square pieces using a sharp razor blade. The sliced leaf pieces were digested in 20 mL protoplast isolation buffer (0.25% Cellulase ONOZUKA R-10, 0.04% Pectinase, 400 mM sorbitol, 20 mM MES-KOH, pH 5.2, 0.5 mM CaCl_2_) and incubated for 4 h at 28 °C in the dark. Digested samples were then filtered with 100 um filter and centrifuged at 110×*g* for 7 min. The collected protoplast was re-suspended in enzyme-free protoplast isolation buffer and centrifuged again. After centrifugation, the supernatant was discarded, and the collected protoplast was used for subsequent experiments.

### Fractionation of chloroplasts

The protocol for isolation and fractionation of chloroplasts was adapted from Rensink et al. (1998), Kieselbach et al. (1998), Tissot et al. (2008), and Lentz et al. (2012). Briefly, intact protoplasts were re-suspended in 4 mL protoplast breaking buffer (300 mM sorbitol, 20 mM tricine-KOH, pH 8.4, 5 mM EDTA, 5 mM EGTA, 10 mM NaHCO_3_, 0.1% BSA). The suspension was transferred into a syringe and filtered through four layers of Miracloth (Millipore, USA) to break the protoplast. The filtered solution was centrifuged at 110×*g* for 7 min, 4 °C, and the chloroplast pellet was re-suspended in 1.6 mL chloroplast lysis buffer (62.5 mM Tris–HCl, pH 5.7, 2 mM MgCl_2_). After incubating on ice for 15 min with gentle vortexing at 5-min intervals, the supernatants (stroma) and pellets (thylakoid) were separated by centrifugation at 7500×*g* for 5 min, 4 °C. The stromal fraction was separated from the supernatant through recentrifugation at 20,000×*g* for 10 min, 4 °C. The thylakoid pellet was washed with each of the following buffers: (I) 10 mM sodium pyrophosphate, pH 7.8; (II) 300 mM sucrose, 2 mM tricine, pH 7.8; and (III) 100 mM sucrose, 50 mM NaCl, 5 mM MgCl_2_, 30 mM sodium phosphate, pH 7.8. Next, the thylakoid pellets were re-suspended in the last buffer and disrupted by sonication on ice for four times for 10 s. Supernatants were obtained by centrifugation of the sonicated sample at 16,000×*g* for 10 min, 4 °C. The lumenal extract was obtained by recentrifugation of these supernatants at 20,000×*g* for 10 min, 4 °C. Thylakoid membranes were washed with the last buffer and centrifuged at 16,000×*g* for 10 min, 4 °C. Finally, thylakoid membranes were re-suspended in the same buffer. These fractions were used for Western blotting.

### Immunoprecipitation and western blots

Immunoprecipitation was performed using GFP-Trap beads (ChromoTek, Germany) with fractionated chloroplast samples following the manufacturer’s instructions. Immunoprecipitation samples were separated by 12% SDS-PAGE gel and subsequently transferred to polyvinylidene fluoride (PVDF) membranes (GE Healthcare, USA) using a wet transfer apparatus (Bio-Rad, USA). The immunoreactive proteins were detected using a primary polyclonal antibody against GFP and LHCII type II chlorophyll a/b-binding protein (Lhcb2, Agrisera, Sweden). Immune signals were detected using Immobilon Western Chemiluminescent HRP substrate (Millipore, USA) following the manufacturer’s instructions and visualized using ImageQuant LAS 4000 mini (GE Healthcare, USA).

### Edman sequencing

Protein samples for electroblotting for Edman sequencing on PVDF membrane were prepared as above. After blotting, PVDF membranes were soaked in distilled water for 5 min and then stained for 5 min with Coomassie Brilliant Blue R-250 reagent (Biosesang, Korea). Stained membranes were destained several times using the destaining solution (40% methanol and 10% glacial acetic acid), thoroughly washed with distilled water, and then air-dried. Protein spots were cut from the PVDF membrane, and then amino acid sequence was determined by sequential Edman degradation using an Automated Edman Sequencer at the Life Science Laboratories (Korea).

## Supplementary Information

Below is the link to the electronic supplementary material.

Electronic supplementary material 1 (DOCX 209 kb)

Electronic supplementary material 2 (DOCX 21 kb)
