# Supplementary material for: The ten amino acids of the oxygen-evolving enhancer of tobacco is sufficient as the peptide residues for protein transport to the chloroplast thylakoid
Source: Plant Mol Biol. 2021 Jan 3;105(4):513–23. doi: 10.1007/s11103-020-01106-8 (PMC7892526; doi:10.1007/s11103-020-01106-8)

Supplementary Figure S1. Amino acid sequence comparison of various chloroplast targeting peptides. (A) The sequence comparison of transit peptide between Tat pathway loading proteins of various plant species. The conserved sequence, RR, for Tat machinery binding residue is indicated in bold letters. (B) The hydropathy value of NtOE23 transit peptides was calculated and indicates hydrophilic and hydrophobic residues.


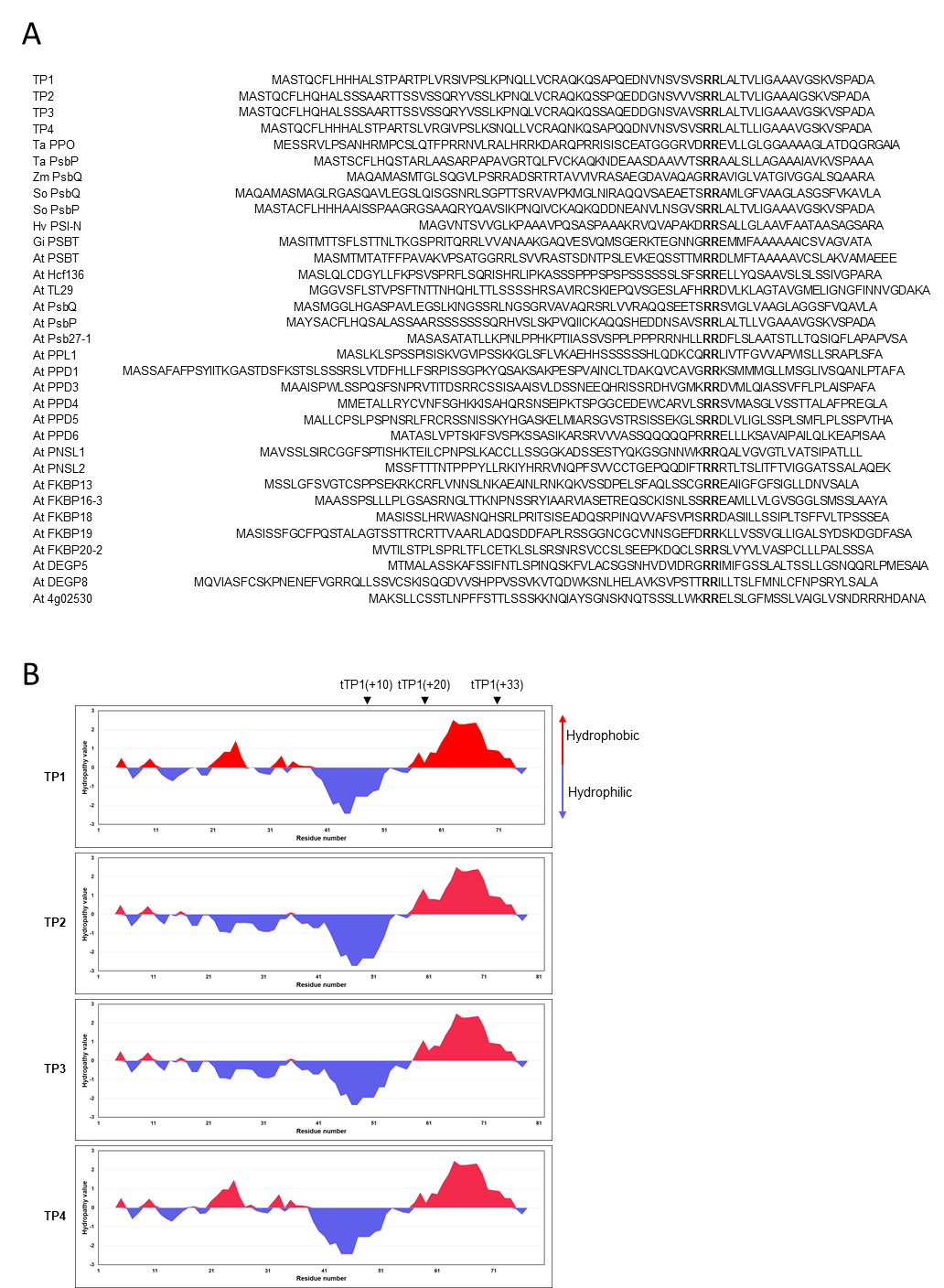

Supplement: Supplementary file 1 — Electronic supplementary material 1 (DOCX 209 kb) [file 11103_2020_1106_MOESM1_ESM.docx]
