# Supplementary material for: The ten amino acids of the oxygen-evolving enhancer of tobacco is sufficient as the peptide residues for protein transport to the chloroplast thylakoid
Source: Plant Mol Biol. 2021 Jan 3;105(4):513–23. doi: 10.1007/s11103-020-01106-8 (PMC7892526; doi:10.1007/s11103-020-01106-8)
Supplement: Supplementary file 2 — Electronic supplementary material 2 (DOCX 21 kb) [file 11103_2020_1106_MOESM2_ESM.docx]

**Supplementary Data**

Supplementary Table S1. Percent identity and similarity of NtOE23 TPs

| **Identity**  **Similarity** | **TP1** | **TP2** | **TP3** | **TP4** |
| --- | --- | --- | --- | --- |
| **TP1** | —  — | 77.1 | 77.1 | 92.5 |
| **TP2** | 81.9 | —  — | 96.3 | 72.6 |
| **TP3** | 81.9 | 97.6 | —  — | 72.6 |
| **TP4** | 95.0 | 81.0 | 81.0 | —  — |

Supplementary Table S2. Percent identity and similarity of NtOE23 TP and other OE23 TP from diverse plant species

| **Species** | **TP1** | **TP2** | **TP3** | **TP4** |
| --- | --- | --- | --- | --- |
| *Capsicum annuum* | 61.7(71.6) | 59.0(67.5) | 57.8(67.5) | 57.3(69.5) |
| *Solanum tuberosum* | 66.7(75.0) | 67.4(70.9) | 67.4(72.1) | 64.7(72.9) |
| *Solanum lycopersicum* | 63.9(74.7) | 67.1(72.9) | 67.1(74.1) | 61.9(72.6) |
| *Glycine max* | 69.6(81.0) | 62.2(72.0) | 63.4(72.0) | 63.8(77.5) |
| *Arabidopsis thaliana* | 53.0(68.7) | 64.6(81.7) | 67.1(82.9) | 48.9(60.0) |
| *Oryza sativa* | 50.0(56.2) | 49.4(60.2) | 49.4(57.8) | 48.1(55.6) |
| *Triticum aestivum* | 45.7(55.6) | 49.4(57.8) | 48.2(57.8) | 45.1(54.9) |
| *Zea mays* | 42.5(57.5) | 38.2(52.8) | 37.9(52.9) | 42.0(56.8) |
| *Sorghum bicolor* | 43.0(55.7) | 45.3(59.3) | 46.5(59.3) | 50.0(58.5) |

* The numbers in parentheses are similarity. All numbers are percent values.

Supplementary Table S3. The primer sets for PCR used in this reports.

| **Prime name** | **Sequence(5'−3')** |
| --- | --- |
| X55354-F | TCTAGAATGGCTTCCACACAATG |
| X55354-R | GGATCCAGCATCAGCAGG |
| X62425-F | TCTAGAATGGCTTCAACACAATGTT |
| X62425-R | GGATCCAGCATCTGCAGG |
| X62427-F | TCTAGAATGGCTTCCACTCAATG |
| X62427-R | GGATCCAGCATCAGCAGG |
| cTP1-R | GGATCCACGGCACACCAATAATTG |
| tTP1(+3)-R | GGATCCTTTTTGGGCACGGCAC |
| tTP1(+10)-R | GGATCCATCTTCTTGGGGAGCAG |
| tTP1(+20)-R | GGATCCTCTACGAGAGACAGA |
| tTP1(+33)-R | GGATCCACCAACGGCAGCAGCA |
